# How does public digital procurement enhance corporate total factor productivity? The role of industry–university–research collaboration

**DOI:** 10.1371/journal.pone.0330160

**Published:** 2025-08-29

**Authors:** Kan Jia, Jinqi Qin, Yang Li

**Affiliations:** 1 School of Management, Zhejiang University of Technology, Hangzhou, China; 2 School of Public Administration, Zhejiang University of Technology, Hangzhou, China; 3 School of Cultural Creativity and Management, Communication University of Zhejiang, Hangzhou, ChinaThese authors contributed equally to this work; Manipal Academy of Higher Education, INDIA

## Abstract

This study innovatively employs large language model (LLM) technology to construct a public digital procurement (PDP) index and, by utilizing microlevel data from Chinese A-share listed companies (2015–2023), systematically examines the impact of PDP on corporate total factor productivity (TFP) and its underlying mechanisms. The results demonstrate that PDP has a significantly positive effect on corporate TFP, and this conclusion remains robust after endogeneity concerns are addressed and multiple robustness tests are conducted. Mechanism analysis reveals that PDP significantly increases corporate TFP primarily through three pathways: enhancing digital technology innovation, alleviating financial constraints, and improving corporate information disclosure. Furthermore, in the context of industry–university–research (IUR) collaboration, PDP has a more pronounced positive effect on corporate TFP. Additional analysis indicates a synergistic effect between PDP and corporate digital transformation, meaning that firms with a higher level of digital transformation can more effectively leverage PDP to achieve TFP growth. This study provides critical theoretical and empirical evidence for leveraging PDP to improve corporate TFP and offers important references for government departments in optimizing the design of PDP policies and enhancing their implementation effectiveness.

## 1. Introduction

With the deepening of a new round of technological revolution and industrial transformation, the scope of public procurement is gradually expanding to cover new technology products and services, including 5G, the industrial internet, the Internet of Things (IoT), artificial intelligence (AI), cloud computing, blockchain, and big data. This shift drives the digital and intelligent transformation of public procurement. Not only does it significantly increase procurement efficiency, but it also facilitates the gradual evolution of traditional procurement targets toward digital products and services with greater technological sophistication. Consequently, it imposes greater demands on the comprehensive capabilities of suppliers [1]. This paper defines this new procurement practice as public digital procurement (PDP), which is a novel practice where the government conducts procurement activities centered on digital infrastructure by publishing various procurement orders, covering digital services, digital products, and their integrated applications [2].

The impact of PDP on enterprises and economic development manifests at multiple levels. First, PDP encourages enterprises to increase their R&D investment through a clear and stable demand orientation and revenue expectations [3], accelerating the pace of technological iteration and diffusion [4,5]. Simultaneously, by optimizing the efficiency of government fund utilization, PDP alleviates corporate financing constraints, improves the corporate financial structure, and increases resource allocation efficiency [2]. Furthermore, when digital procurement platforms are leveraged, PDP significantly increases the transparency and fairness of the procurement process, which not only mitigates information asymmetry [6]but also improves organizational coordination efficiency [7].

Although existing research has preliminarily explored the impact of PDP on different dimensions of enterprise development, the literature focuses primarily on corporate digital transformation and innovation activities, leaving a gap in systematic explanations of whether and how public procurement increases overall corporate total factor productivity (TFP). As a core indicator of an economy’s long-term growth potential and sustainable development capacity, TFP fundamentally captures the overall productive efficiency achieved by enterprises in comprehensively utilizing factor inputs such as labor, capital, and land [8]. In the context of challenges including waning demographic dividends, rising labor costs, and increasingly stringent resource and environmental constraints [9], sustainable corporate development depends critically not only on the continued accumulation of production factors but also, more crucially, on the sustained improvement in TFP.

The literature shows that government support plays an indispensable and critical role in increasing corporate TFP. On the one hand, government subsidies effectively incentivize innovation activities that ultimately translate into corporate TFP growth by alleviating financing constraints for corporate R&D investment and sharing innovation risks [10,11]. On the other hand, government procurement can influence corporate TFP through channels such as creating stable demand, fostering competition, and promoting knowledge diffusion [12]. However, specifically in the digital domain, how PDP systematically drives corporate TFP improvement still requires deeper theoretical and empirical research.

China provides a suitable context for studying this issue. With the rapid development of its digital economy, the Chinese government is vigorously promoting digital infrastructure construction, and the scale of public digital procurement continues to expand. Public data show that in 2024, China’s digital infrastructure industry reached a market size of CNY 614.4 billion, increasing by 30.8%. The China Public Procurement Development Report indicates that in 2020, national government procurement totaled CNY 3,697.06 billion, accounting for 10.2% of fiscal expenditure that year, with the digital transformation of public procurement accelerating. In light of this trend, we employed natural language processing techniques to accurately identify all public digital procurement contracts awardeda list of A-share listed enterprises. On this basis, we conducted panel regression analyses and empirically found that PDP significantly enhances corporate TFP, with digital technology innovation, the alleviation of financing constraints, and information disclosure serving as partial mediators. Furthermore, the positive impact of PDP on TFP is amplified by industry–university–research (IUR) collaboration.

Overall, our study makes the following contributions: (i) While previous research has preliminarily explored the effects of digital government policies on aspects such as corporate investment acquisition [13], it has yet to systematically quantify the actual implementation intensity of government policies. This study is the first to employ large language model (LLM) to innovatively construct a precise PDP index, thus accurately quantifying and evaluating local governments’ fiscal expenditures on digital products/services and their economic utility. (ii) Existing studies predominantly focus on supply-side determinants of corporate TFP such as human capital and R&D investment [10,14]. This study explicitly adopts a demand-side perspective to reveal how PDP catalyzes corporate TFP growth. (iii) This study further uncovers the internal mechanisms through which PDP affects corporate TFP. We clearly identify three mediating mechanisms: the digital technology innovation effect, the financial constraint alleviation effect, and the corporate information disclosure effect.

The paper is structured as follows: The subsequent section presents a review of the pertinent theoretical foundations and formulates our research hypotheses concerning the impact of PDP on corporate TFP. Section 3 presents the model specification, variable description, and descriptive analysis. Sections 4 and 5 provide the empirical findings and a series of statistical tests. Finally, the conclusions, limitations, and future research directions are discussed.

## 2. Literature review and research hypotheses

### Public digital procurement and corporate total factor productivity

Public procurement refers to the process whereby governments acquire goods or services through tendering procedures using public funds [15]. Owing to its role in allocating public resources, it has long been regarded as a policy tool for increasing economic efficiency. Specialized procurement subtypes (e.g., public green procurement, public innovation procurement) demonstrate greater effectiveness than traditional contracts [16].

Driven by the digital economy wave, public procurement has progressively increased acquisitions of digital products/services, giving rise to PDP. Digital infrastructure has become a critical driver of socioeconomic development globally, especially in China [12]. Traditional supply-side policies exhibit limitations in addressing digital economy challenges [15], prompting governments to intensify digital infrastructure procurement. Against this backdrop, we define PDP as government tenders for digital infrastructure construction, encompassing digital services, products, and integrated applications.

Existing research examines the impacts of PDP on enterprises along three dimensions. First, organizational coordination studies show that digital infrastructure investment significantly reduces communication costs, enhances coordination capabilities, and mitigates information asymmetry [6]. Furthermore, by providing stable demand, PDP strengthens strategic orientation and resource allocation capabilities while alleviating financing constraints, thus driving digital transformation [2]. Second, technological innovation research indicates that public procurement promotes cost reduction and innovation through standardization/modularization [4,5]. Finally, financial security studies demonstrate that PDP creates stable market expectations that incentivize private R&D investment while reducing operating costs and optimizing supply chains [7]. Despite these insights, the literature overlooks the systematic impact of PDP on corporate productivity.

Corporate TFP determines the long-term growth potential of economies and sustainable development foundations. Internally, human capital, R&D investment, and digital technology adoption drive TFP improvement [14,17,18].

However, TFP improvement relies more crucially on external policy synergy than on internal capabilities [19]. Government interventions effectively reduce information asymmetry, lower technology diffusion barriers, and optimize funding chains, directly boosting TFP.

Specifically, PDP’s digitalization enhances transparency/efficiency and diversifies suppliers through streamlined registration. Doing so creates market access for small and medium-sized enterprises (SMEs), especially those in remote areas, enabling equitable competition with large firms via e-platforms [11]. Intensified competition incentivizes operational scaling. Additionally, PDP strengthens enterprise–supply chain–government connectivity, facilitating data flow. E-government platforms empower efficient communication (e.g., via blockchain), improving digital technology application and enabling data-driven decisions. Finally, PDP deepens public‒private symbiosis [15], providing financial support through “relational performance” (mutual value creation via institutional collaboration) and governmental endorsement that reduces financing costs [20].

Therefore, we can formulate the following hypothesis:

**Hypothesis 1: PDP has a positive effect on corporate TFP.**

### Mechanism analysis

#### Promoting digital technology innovation.

PDP can increase corporate TFP through innovation incentives. According to demand-induced innovation theory, the stringent requirements and promising market prospects of public digital projects fuel a demand for high-tech components and digital core technologies, thereby stimulating digital technological advancements. PDP provides essential conditions for corporate innovation, including opportunity identification and technical knowledge exchange.

Within the PDP process, government entities begin by outlining the requirements for digital economy products. This is followed by a competitive selection process, encompassing tenders, negotiations, and consultations, to ascertain a suitable supplier with the capacity to produce. This process elucidates the market demand for digital technologies, particularly the diversified and personalized demands of end-consumer markets in the digital economy. This, in turn, facilitates a reduction in information search and opportunity identification costs for corporate innovation and minimizes uncertainties in the R&D process. Moreover, PDP necessitates the use of digital technologies such as big data, cloud computing, and AI, thereby emphasizing knowledge exchange and interaction. Contracts forged under PDP foster a reciprocal and synergistic partnership between government agencies and enterprises. This collaboration facilitates the exchange of demand information, expert knowledge, and critical inputs such as data, ultimately reducing opacity in the R&D process [21]. Ultimately, enterprises can benefit from PDP projects when new technologies are developed. By reducing uncertainty and opacity in R&D processes, PDP enhances corporate innovation capacity, leading to the advancement of existing technological capabilities.

As the level of digital technology continues to rise, enterprises can more accurately grasp market demands, optimize production processes, improve product quality and effectively reduce operating costs; these positive changes collectively increase corporate TFP [22].

Thus, the current study proposes the following

**Hypothesis 2: Digital technology innovation mediates the relationship between PDP and corporate TFP.**

### Alleviating financial constraint

Corporate competitive advantage stems from unique resources and capabilities, with capital as a crucial resource supporting enterprise development and profitability [23]. Enterprises often resort to external financing to alleviate cost pressures. China’s current financing system is predominantly bank-led. However, enterprises often face unstable early-stage returns and significant profit volatility [8], coupled with information asymmetry in the financing sector, leading to tight financing constraints [24].

According to asymmetric information theory, where the existence of unequal information between market participants can lead to inefficiencies and adverse selection, sellers often have more information about the quality of their products than do buyers, which can lead to potential market failure [25]. Completing technologically advanced and highly competitive PDP projects strengthens a company’s market position, conveys positive signals to external investors about the enterprise’s technological prowess and market potential, and overall operational health, which potentially attracts more external capital inflows and reduces credit costs associated with corporate financing, thereby easing financing constraints [6].

The alleviation of financing constraints provides enterprises with more stable and sustainable funding sources, enabling increased long-term investment in technological R&D, equipment upgrades, process optimization, and human capital. This not only increases resource allocation efficiency and production organization efficiency but also strengthens corporate flexibility and adaptability in response to market fluctuations. Consequently, it reinforces innovation capabilities and operational resilience, thereby effectively driving sustained growth in corporate TFP.

Thus, the current study proposes the following

**Hypothesis 3: Financial constraint alleviation mediates the relationship between PDP and corporate TFP.**

### Enhancing information disclosure

Information asymmetry theory profoundly reveals that incomplete information distribution in capital markets creates significant informational gaps between participants (e.g., investors and firms, owners and managers), potentially triggering adverse selection and moral hazard. Consequently, corporate managers must proactively disclose information to mitigate such asymmetries [25].

PDP increases corporate information disclosure practices and alleviates multidimensional information asymmetries. In the regulatory dimension, government procurement contracts impose compulsory disclosure clauses requiring suppliers to publish core information on their technical capabilities, project execution standards, and digital security compliance, substantially increasing corporate transparency [26]. In the market competition dimension, to gain competitive advantage in PDP tenders, firms possess strong intrinsic incentives to voluntarily augment disclosures. They may release detailed financial reports, technical white papers, project implementation plans, or social responsibility reports to signal comprehensive capabilities in R&D, project management, and regulatory compliance to potential investors, financial institutions, and regulators [27], thereby strengthening their PDP market competitiveness.

Information disclosure drives corporate TFP growth through synergistic capital allocation optimization and increased governance efficacy. From a capital allocation perspective, high-quality disclosures correct investor misvaluation of digital assets and technological potential, reducing equity financing costs and increasing stock liquidity caused by information asymmetry [28]. This redirects capital toward high-efficiency projects, optimizing factor combination efficiency. From a corporate governance perspective, robust disclosure intensifies the oversight of managerial investment decisions, constraining self-serving overinvestment on the basis of informational advantages. It redirects resources to high-return areas such as innovation R&D and compels continuous optimization of internal resource allocation, unlocking the potential of organizational factor reallocation to sustainably increase TFP [29].

Thus, the current study proposes the following

**Hypothesis 4: Information disclosure plays a pivotal mediating role in the positive relationship between PDP and corporate TFP.**

### The enhancing effect of industry–university–research collaboration

IUR cooperation, as a mechanism for promoting innovation and productivity, holds particular significance in the digital era. By pooling resources and expertise from industry, academia, and research institutions, IUR collaboration not only accelerates the R&D and commercialization of technological achievements but also increases corporate technological capabilities and resource allocation efficiency by facilitating knowledge spillovers, technology transfer, and collaborative innovation, thereby driving growth in corporate TFP [30]. In the context of digital public procurement, IUR cooperation can act as a powerful catalyst for increasing TFP. By involving universities and research institutions in public projects, governments not only gain access to a broader range of innovative solutions and expert knowledge but also help bridge the gap between research and commercialization, ensuring that cutting-edge technologies are rapidly and effectively applied in practice [31].

Moreover, IUR cooperation fosters close collaboration between buyers and suppliers, facilitating the transfer of knowledge and technology and further amplifying the positive impact of digital public procurement on TFP. Restricted by several institutional and structural constraints, the commercialization of university-based technology is inefficient in China [32]. This leads to a phenomenon where the output rate of innovation achievements from universities and research institutions is high but the conversion rate to practical applications is low. This issue stems from the lack of adequate policy guidance, incentives, and safeguards in the industrialization and marketization of technological achievements. Securing public procurement bids in the digital realm often necessitates advanced technological capabilities, prompting many enterprises to seek collaborations with academic and research institutions. These partnerships are driven by the high digital technology requirements typically associated with digital service or product-related orders. Compared with enterprises with similar characteristics, equipment suppliers tend to introduce more radical product innovations [33]. This finding underscores the potential for knowledge transfer and innovation stimulation through IUR collaboration. Collaborations with universities have been shown to be instrumental in ensuring the efficient completion of orders.

Therefore, the research hypotheses are as follows:

**Hypothesis 5: IUR collaboration has a positive effect on corporate TFP.**

**Hypothesis 6: IUR collaboration positively enhances the relationship between PDP and corporate TFP.**

**Fig 1:**
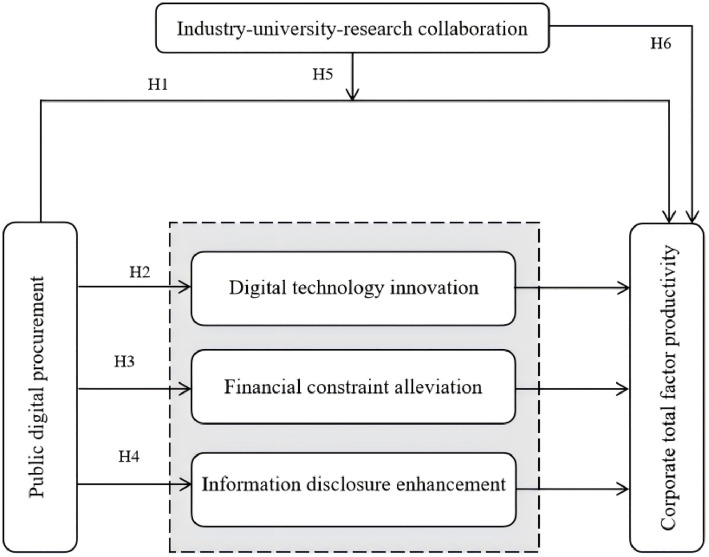
The mechanism of PDP and corporate TFP.

A conceptual model of the study is proposed in Fig 1.

## 3. Methodology

### Model specification

#### Baseline model and baseline model.

To identify the impact of PDP on corporate TFP, this paper constructs the following two-way fixed effects models:


$$TFP_{it}=\alpha_{0}+\alpha_{1}PDP_{it}+\mu_{i}+\theta_{t}+Control_{it}+\varepsilon_{it}$$ (1)

#### Benchmark mediating effect model.

To analyze the mediating effects of three potential mediators—digital technology innovation, financial constraint alleviation, and information disclosure enhancement—on the relationship between PDP and corporate TFP, we utilize a three-step approach: first, we regress Eq. (1) to verify the influence of PDP on TFP; second, we regress Eq. (2) to test the influence of PDP on each mediator. Finally, Eq. (3) is regressed to further test the simultaneous influence of the independent and mediating variables on the dependent variables.


$$TFP_{it}=\alpha_{0}+\alpha_{1}PDP_{it}+\mu_{i}+\theta_{t}+Control_{it}+\varepsilon_{it}$$ (2)

$$M_{it}=\gamma_{0}+\gamma_{1}PDP_{it}+\mu_{i}+\theta_{t}+Control_{it}+\varepsilon_{it}$$ (3)

$$TFP_{it}=\delta_{0}+\delta_{1}M_{it}+\delta_{2}PDP_{it}+\mu_{i}+\theta_{t}+Control_{it}+\varepsilon_{it}$$ (4)

#### Benchmark moderating effect model.

To further estimate the moderating effect of IUR collaboration on the relationship between PDP and corporate TFP, this paper constructs the following two-way fixed effects models:


$$TFP_{it}=\beta_{0}+\beta_{1}PDP_{it}+\beta_{2}IUR_{it}+\beta_{3}PDP_{it}*IUR_{it}+\mu_{i}+\theta_{t}+\beta_{4}Control_{it}+\varepsilon_{it}$$ (5)

Where subscripts *i* and *t* represent the enterprise fixed effects and time fixed effects, respectively; $TFP_{it}$ is defined as a measure that reflects the efficiency of production and the technological level of an enterprise; $PDP_{it}$ is defined as a measure that reflects the scale of PDP contracts obtained by the enterprise in the previous period; and $M_{it}$ is a mediator variable, where each *M* represents DT, SA, and KV. $Control_{it}$ is a set of control variables; $\mu_{i}$ represents enterprise fixed effects; $\theta_{t}$ represents time fixed effects; and $\varepsilon_{it}$ is the random error term. In Eq. (1), the coefficient $\alpha_{1}$ captures the direct effect of PDP on TFP. When $\alpha_{1}$ is greater than zero, PDP promotes corporate TFP. When $\alpha_{1}$ is less than zero, PDP inhibits corporate TFP. Eq. (2) tests the effect of PDP on each mediator $M_{it}$, with coefficients $\gamma_{1}$ indicating the strength of this relationship. The final Eq. (3), incorporating the mediators, allows us to assess the indirect effects of PDP on TFP through each mediator. If a significant reduction in the absolute value of $\delta_{2}$ compared with $\alpha_{1}$, along with a significant $\delta_{1}$, it suggests mediation through $M_{it}$. In Eq. (4), the coefficient $\beta_{3}$ captures the interaction effect between PDP and IUR on TFP.

### Measurement

#### Explanatory variable.

Referring to the method of [4], we selected PDP as the explanatory variable and employed natural language processing methods to construct the PDP variable. LLM possess robust contextual semantic comprehension capabilities and generalized linguistic knowledge acquired from massive unannotated texts. Through transfer learning and fine-tuning, such models efficiently adapt to domain-specific classification tasks, significantly increasing recognition accuracy for feature-specific terms in complex unstructured texts [34]. Leveraging the scientific rigor and advanced performance of LLM in text representation and classification, we constructed feature lexicons and executed classification via the following procedure.

**Fig 2:**
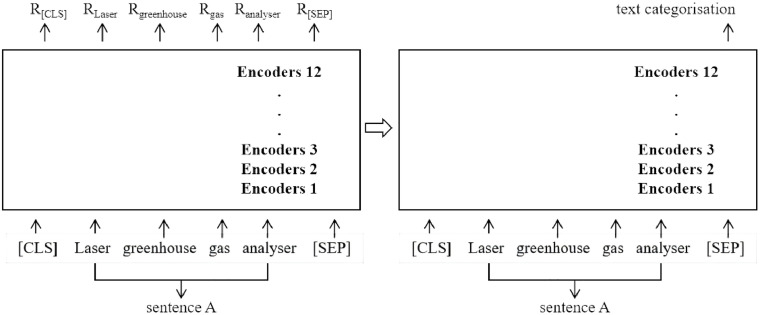
Pretrained LLM model architecture.

(i) We used Python to crawl all public procurement contracts from the China Government Procurement Network (2015–2023), extracting key information, including project names, purchasers, suppliers, subject matter descriptions, specifications/service requirements, contract values, and dates. (ii) We subsequently match the supplier names in the procurement orders with the names of A-share listed companies and their subsidiaries, successfully matching 101,373 orders and clearing the data for further processing. (iii) To develop a digital procurement lexicon, we adopted the approach of [4,35], utilizing the Jieba word segmentation tool to tokenize documents such as the “Statistical Classification of the Digital Economy and Its Core Industries”. Adverbs, conjunctions, and other nonsubstantive function words were removed from the segmentation results. The top 200 high-frequency substantive words were selected by descending frequency to form a base lexicon, which was then expanded through LLM-driven semantic association capabilities for synonym generalization and contextual broadening, ultimately yielding a lexicon of over 400 digital economy keywords. (iv) We then match this digital-related keyword library with public procurement text information. If a project contract name, main subject name, specifications, or service requirements contained these keywords, then it was identified as a digital procurement project. To further improve identification accuracy, following [36], we optimized the matching process via LLM fine-tuning: 1,500 data points were randomly selected for manual annotation (two researchers independently labeled records with a kappa coefficient≥0.8), and a pretrained LLM was fine-tuned using masked language modeling and next sentence prediction (the model architecture is shown in Fig 2). Dynamic learning rate scheduling optimized the classification layer parameters, achieving 90% accuracy on the test set. (v) We aggregate the data by firm and year, and the total amount of PDP contracts for each listed company is calculated annually and standardized according to the company’s total assets: *PDP = ln(PDP contract amount+1)/ln(corporate total assets)*. Through this process, 526 observations of PDP data were ultimately obtained.

#### Explained variable.

In this study, we employed corporate TFP as the dependent variable. TFP is a comprehensive indicator that measures corporate production efficiency given a set of input factors. It reflects the contributions of various factors, such as technological progress and management efficiency improvements, to corporate output [37] Among the methods for estimating TFP, the Olley–Pakes (OP) and Levinsohn–Petrin (LP) approaches are particularly prominent [38,39]. However, the OP method may result in a substantial loss of observations, especially when the sample contains instances of zero investment. To overcome this limitation, we chose the LP method to measure TFP in emerging economies. The fundamental idea of the LP method is to use intermediate inputs as a proxy for TFP. This approach allows researchers to select proxy variables on the basis of available data, thereby minimizing data loss. The baseline Cobb‒Douglas production function for calculating TFP is presented as follows:


$$\ln Y_{it}=\gamma_{0}+\gamma_{1}\ln L_{it}+\gamma_{2}\ln K_{it}+\gamma_{3}\ln I_{it}+\ln TFP_{it}+\varepsilon_{it}$$

Where Y represents output; L denotes labor input; K represents capital input; I denotes intermediate input, measured by cash paid for goods and services; ϵ is the error term; and subscripts i and t denote enterprise and time, respectively. In our empirical estimation, these variables are operationalized as follows: total output is represented by operating income, capital input by net fixed assets, labor input by the number of employees, and intermediate input by cash paid for goods and services received. To ensure the robustness of our results, we also employed the OP method to recalculate corporate TFP in the latter part of this study [40].

#### Mediator variables.

For digital technology, we utilize the digital-related keyword library constructed in the previous sections to identify digital technology innovation patents applied for by enterprises, aggregating the identified patents from the “enterprise-year” dimensions to construct a measurement indicator DT for digital technology innovation at the enterprise level. The specific formula used is *DT = ln (total number of DT invention patent applications* *+* *1).*

For financial constraints, we calculate the level of financial constraints using firm size and firm age. The specific formula is as follows: *SA = –0.737 × size + 0.043 × size*^*2*^ *– 0.04 × age*. This index generally takes negative values, with a larger absolute value indicating a greater degree of financial constraint.

For information disclosure, we employ the KV index to capture the extent and quality of corporate information disclosure, which reflects the degree of information asymmetry from the perspective of investors. The index is calculated via the following equation:


$$\begin{array}{c} \ln(P_{t}-P_{t-1})/P_{t-1}=\sigma_{0}+\sigma_{1}\left(\frac{Vol_{t}}{Vol_{0}}-1\right)\varepsilon \end{array}$$

Where $P_{t}$ denotes the stock closing price on day *t,* $Vol_{t}$ is the trading volume on day *t,* and $Vol_{0}$ represents the average daily trading volume over the sample period.

#### Moderator variable.

IUR collaboration, following the approach of [33], was measured through joint patent applications. The specific procedure is outlined as follows. We collected patent application data for all listed companies from the website of the China National Intellectual Property Administration. Through keyword searches, we screened the applicant information of these patents and defined invention patents jointly applied for by listed companies with universities, research institutes, or research centers as IUR collaboration patents. On the basis of these collaboration patents, we then determine whether a listed company engages in IUR collaboration, assigning a value of 1 if it does, and 0 otherwise.

#### Control variables.

$Control_{it}$ represents the vector of control variables. Following the research of [41,10], we included the following corporate control variables to reduce the possibility of estimation bias in the coefficient of the key variable: Cashflow, Lev, Growth, Board, TobinQ, and Cap.

### Sampling

This study focuses on Chinese A-share listed companies from 2015 to 2023. A pivotal change in 2015 was the mandate to increase the transparency of public procurement information, which facilitated better disclosure of related contract details and enabled us to access data on PDP. Specifically, PDP data were obtained from the Chinese Government Procurement Network; corporate data were extracted from the CSMAR database, annual reports of listed companies, the China National Intellectual Property Administration website, and the China Statistical Yearbook. To ensure sample quality and the reliability of our results, the following sample selection criteria were applied: (i) companies that were subject to special treatment (ST) or other risk warnings (ST) during the study period were excluded, (ii) financial companies were removed, and (iii) observations with missing data for key variables were eliminated. After screening, a panel dataset comprising 26,306 enterprise-year observations was obtained. To mitigate the impact of extreme values, all continuous variables at the 1st and 99th percentiles were winsorized. To control for serial correlation at the enterprise level, cluster-robust standard errors were applied in all regression analyses.

### Descriptive statistics

The descriptive statistics associated with the variables are provided in Table 1. For PDP, the mean value is 0.007, with a standard deviation of 0.052. This finding indicates that the overall scale of PDP is relatively small and that there is considerable variation in the extent to which different firms secure public digital orders. For TFP, the mean value is 6.743, with a standard deviation of 0.834. The sample captures sufficient variation to explore the relationship between PDP and corporate TFP. Furthermore, other variables also demonstrate notable variations across firms, attesting to the good distribution of the sample.

**Table 1:** Statistical description of the variables.

Variables	Definition	Obs	Std. Dev.	Min	Mean	Max
**TFP**	Corporate total factor productivity	26049	0.834	3.447	6.743	11.447
**PDP**	Public digital procurement	26049	0.052	0	0.007	0.793
**DT**	Digital technology level	26049	1.177	0	0.604	6.885
**SA**	Financial constraints SA index	26049	0.247	−5.728	−3.895	−2.563
**KV**	Corporate disclosure KV index	26004	0.203	0.001	0.535	2.033
**IUR**	Industry–university–research collaboration	26049	0.078	0	0.006	1
**DIG**	Digitalization	26049	1.303	0	1.285	6.211
**Lev**	Leverage ratio	26049	0.196	0.049	0.409	0.924
**Cashflow**	Cash flow ratio	26049	0.066	−0.172	0.051	0.266
**Growth**	Revenue growth rate	26049	0.377	−0.653	0.143	3.808
**Board**	Board size	26049	0.194	1.609	2.099	2.708
**TobinQ**	Tobin’s Q value	26049	1.402	0.789	2.087	16.647
**Cap**	Capital asset	26049	0.846	12.636	14.633	17.597

## 4. Empirical results and discussion

### Results of the baseline model

Table 2 presents the baseline regression results of our study. Models 1–4 showcase the stepwise regression outcomes, progressively incorporating year fixed effects, firm fixed effects, and finally both year and firm fixed effects. The results of Model 4 reveal that the coefficient of the explanatory variable PDP is 0.128 and significant at the 1% level. The R² value suggests that our model accounts for a substantial portion of the factors influencing corporate TFP. A baseline coefficient of 0.128 implies that a one-unit increase in the standardized PDP index leads to a 0.128 unit increase in the logarithm of TFP. Given that the average TFP in our sample is 6.743 with a standard deviation of 0.834, this effect translates to an approximate calculated percentage, calculated as a 15.35% increase in terms of the standard deviation of TFP. This indicates an economically significant impact.

**Table 2:** Baseline regression results.

	Model 1	Model 2	Model 3	Model 4
**PDP**	0.468^***^ (0.075)	0.421^***^ (0.075)	0.219^***^ (0.039)	0.128^***^ (0.039)
**Cashflow**	2.577^***^ (0.060)	2.563^***^ (0.061)	1.048^***^ (0.058)	0.953^***^ (0.057)
**Lev**	1.493^***^ (0.021)	1.503^***^ (0.021)	0.469^***^ (0.054)	0.284^***^ (0.052)
**Growth**	0.277^***^ (0.010)	0.278^***^ (0.011)	0.246^***^ (0.010)	0.273^***^ (0.009)
**Board**	0.278^***^ (0.020)	0.299^***^ (0.021)	−0.001 (0.036)	0.071^**^ (0.034)
**TobinQ**	−0.080^***^ (0.003)	−0.076^***^ (0.003)	−0.042^***^ (0.005)	−0.020^***^ (0.005)
**Cap**	0.401^***^ (0.005)	0.397^***^ (0.005)	0.529^***^ (0.019)	0.378^***^ (0.021)
**cons**	−0.327^***^ (0.082)	−0.317^***^ (0.082)	−1.187^***^ (0.292)	0.912^***^ (0.316)
**Obs**	26049	26049	25551	25551
**R** ^**2**^	0.428	0.431	0.899	0.910
**Firm effects**	NO	NO	YES	YES
**Year effects**	NO	YES	NO	YES

*Notes:* The t statistics in parentheses are based on robust standard errors clustered at the firm level. ^*^, ^**^, and ^***^ represent significance levels of 0.10, 0.05, and 0.01, respectively.

To address potential multicollinearity, we calculated the correlation coefficients among the independent variables and obtained the variance inflation factors (VIFs). The mean VIF across all variables is 1.06, with all individual VIF values remaining below 1.5. Consequently, multicollinearity does not pose a significant issue for our regression analysis. Overall, our results support H1, suggesting that PDP significantly encourages enterprises to increase their TFP.

### Endogeneity tests

#### Instrumental variable approach.

To mitigate the potential reverse causality between PDP and corporate TFP, following the research of [4], we employed the city-level annual total number of digital procurement orders as an instrumental variable for enterprises awarded digital procurement. This instrument was chosen on the basis of two considerations. First, a higher initial proportion of PDP orders among listed companies in a city is likely to make it easier for enterprises in that city to obtain such orders, thereby satisfying the relevance requirement. Second, PDP orders received by a city are not directly influenced by the TFP levels of individual enterprises, thereby meeting the exclusion restrictions.

The first-stage regression results are presented in Model 1 of Table 3. The coefficient of IV is 0.024, which is positive and significant at the 1% level, indicating a strong positive correlation between PDP and IV. In the weak instrument test, the F statistic is 2461.56, which far exceeds the critical value, further confirming the validity of the IV. The second-stage regression results are shown in Model 2 of Table 3. The coefficient of PDP is 0.143, which is positive and significant at the 1% level, verifying the robustness of our baseline results.

**Table 3:** Endogeneity test results.

Variables	Model l	Model 2	Model 3	Model 4	Model 5
**PDP**	**TFP**	**TFP**	**PDP_dum**	**TFP**
**PDP**		0.143^***^ (0.041)	0.130^***^ (0.039)		
**IV**	0.024^***^ (0)				
**PDP_dum**					0.042^***^ (0.014)
**Z**				26.282^***^ (0.965)	
**IMR**					−0.144^**^ (0.068)
**Obs**	25551	25551	16378	26049	25551
**R** ^**2**^		0.253	0.911	0.155	0.910
**F-statistic**	2461.56				
**Controls**	YES	YES	YES	YES	YES
**Firm effects**	YES	YES	YES	YES	YES
**Year effects**	YES	YES	YES	YES	YES

*Notes:* The t statistics in parentheses are based on robust standard errors clustered at the firm level. ^*^, ^**^, and ^***^ represent significance levels of 0.10, 0.05, and 0.01, respectively.

#### Propensity score matching.

To address potential sample selection bias, in this research, we employed the propensity score matching (PSM) method. PSM is primarily used to mitigate selection bias caused by observable characteristics, allowing for a more accurate estimation of the impact of PDP on corporate TFP. It creates a dummy variable based on whether an enterprise receives PDP and uses this variable as the treatment variable for PSM radius matching with a caliper of 0.05. We then estimate the effect of PDP on corporate TFP via the matched sample.

The PSM estimation results are presented in Model 3 of Table 3. The results show that in the matched sample, receiving PDP still has a significant positive effect on corporate TFP. On average, enterprises that receive PDP have a TFP that is 0.130 higher than those that do not, which is significant at the 1% level, thereby meeting the exclusion restrictions.

#### Heckman selection model.

To address potential self-selection issues in the model, we employed the Heckman selection model. In the first stage, we used a probit model to estimate the probability of an enterprise receiving PDP, i.e., PDP_dum. We subsequently included the industry-specific mean of PDP as an exogenous exclusion variable in the regression model, thus obtaining the inverse Mills ratio (IMR). The regression results are shown in Model 4 of Table 3, where the Z value is 26.281 and is statistically significant at the 1% level. In the second stage, considering possible sample selection bias, where the government may prefer to award digital procurement projects to enterprises with strong DT innovation capabilities, we introduced the IMR into the baseline regression model. The results of Model 5 in Table 3 indicate that after sample self-selection is considered, the coefficient of PDP_dum is 0.042, which is positive and significant at the 1% level, thereby meeting the exclusion restrictions.

In conclusion, the results of our endogeneity analyses using IV, PSM, and Heckman selection models consistently support our baseline finding, i.e., that PDP significantly increases corporate TFP. The consistency across these multiple methodologies strengthens the reliability of our results.

### Robustness tests

#### Alternative measures of digital public procuring.

In this research, we employed two alternative approaches to measure our key explanatory variable: (i) PDP1, defined as the natural logarithm of the PDP contract amount plus one; and (ii) PDP2, a dummy variable assigned a value of 1 if the enterprise received PDP contracts and 0 otherwise. Models 1–2 in Table 4 report the regression results when these alternative measures are used. Upon substituting PDP with PDP1 and PDP2 in the regression analyses, the coefficients obtained were 0.006 (p < 0.01) and 0.042 (p < 0.01), respectively. Regardless of the measurement approach, the impact of PDP on corporate TFP remains significantly positive, which is consistent with our baseline results.

**Table 4:** Robustness check results.

Variables	Model 1	Model 2	Model 3	Model 4	Model 5	Model 6	Model 7
	TFP	TFP	TFP_OP	TFP	TFP	TFP	TFP
**PDP1**	0.006^***^ (0.002)						
**PDP_dum**		0.042^***^ (0.014)					
**PDP**			0.179^***^ (0.045)			0.128^**^ (0.045)	0.128^**^ (0.048)
**PPnd**				−0.013 (0.015)			
**PPtt**					−0.013 (0.015)		
**R** ^**2**^	0.910	0.910	0.914	0.910	0.910	0.910	0.910
**Controls**	YES	YES	YES	YES	YES	YES	YES
**Firm effects**	YES	YES	YES	YES	YES	YES	YES
**Year effects**	YES	YES	YES	YES	YES	YES	YES
**Industry clustering**	NO	NO	NO	NO	NO	YES	NO
**Regional clustering**	NO	NO	NO	NO	NO	NO	YES

*Notes:* The t statistics in parentheses are based on robust standard errors clustered at the firm level. ^*^, ^**^, and ^***^ represent significance levels of 0.10, 0.05, and 0.01, respectively.

#### Alternative measure of total factor productivity.

To test the sensitivity of our results to TFP calculation methods, we reestimate enterprise TFP via the OP method as an alternative dependent variable. Model 3 in Table 4 presents the corresponding regression results. The coefficient of PDP on OP-estimated TFP remains significantly positive at 0.179, which is significant at the 1% level, thereby reinforcing our main findings.

#### Consideration of other public procurements.

To further corroborate our conclusions, we examined the public procurement of other products from enterprises. Theoretically, if the observed increase in enterprise TFP is indeed driven by PDP, then significant TFP changes should not be observed when the public procures other products. We first aggregate all public procurement orders for each enterprise, scaled by enterprise size (PPtt), and then calculate nondigital procurement contracts, similarly scaled by enterprise size (PPnd). Models 4 and 5 in Table 4 report the regression results for these variables. The regression coefficients for both PPtt and PPnd are −0.013, and neither is statistically significant. The results indicate that public procurement of other product categories does not significantly promote enterprise TFP, further confirming the results of the baseline model.

#### Clustered standard errors test.

To assess the model’s sensitivity to potential within-group correlations, we implemented clustered standard error adjustments along industry and region dimensions, building upon firm and year fixed effects. This approach addresses the likelihood that firms within the same industry or region experience similar policy shocks or external environments, as neglecting such correlations may underestimate standard errors and compromise statistical inference reliability. Model 6 reports estimates with industry-clustered standard errors, showing a PDP coefficient of 0.128, which is statistically significant at the 1% level. Model 7 reports region-clustered standard error estimates, with the PDP coefficient remaining at 0.128 and statistically significant at the 5% level. These results indicate that the productivity-enhancing effect of PDP on corporate TFP is not biased by industry commonalities or regional heterogeneity, further validating the robustness of our findings.

By altering the explanatory and dependent variables, considering other factors such as public procurement, and incorporating clustered standard error tests, the results indicate that the positive relationship between PDP and corporate TFP remains robust. Once again, the results suggest that our findings are unlikely to be a result of instability.

## 5. Mechanism analysis

### The effect of digital technology innovation

Table 5 provides the results of the regression analysis testing the mediating role of DT in the relationship between PDP and TFP. We conduct hierarchical regression analysis in three steps. In column (1), the results reveal a significantly positive relationship between PDP and DT ($\gamma_{1}$ =0.521, p < 0.05). In addition, controlling for PDP, DT must have a significant effect on TFP, and the main effect of PDP should decrease substantially. In column (2), TFP has a significantly positive coefficient for DT ($\gamma_{1}$ =0.05, p < 0.01), and the magnitude of the coefficient for PDP ($\delta_{1}$ =0.125, p < 0.01) decreases compared with the coefficient in the baseline regression ($\delta_{1}$ =0.126, p < 0.01). We conduct a Sobel test to assess the magnitude of the mediating effect, resulting in a Z value of 11.82, which is significant at the 1% level. Therefore, H2 is supported.

**Table 5:** Mechanistic analysis results.

Variables	Digital Technology Innovation		Financial Constraint Alleviation		Corporate Disclosure	
	(1)DT	(2)TFP	(3)SA	(4)TFP	(5)KV	(6)TFP
**PDP**	0.521^***^ (0.218)	0.125^***^ (0.039)	−0.024^***^ (0.007)	0.120^***^ (0.039)	0.073^***^ (0.027)	0.112^***^ (0.039)
**DT**		0.005^***^ (0.002)				
**SA**				−0.303^***^ (0.112)		
**KV**						0.202^***^ (0.014)
**Obs**	25551	25551	25551	25551	25504	25504
**R** ^**2**^	0.584	0.910	0.973	0.911	0.422	0.912
**Controls**	YES	YES	YES	YES	YES	YES
**Firm effects**	YES	YES	YES	YES	YES	YES
**Year effects**	YES	YES	YES	YES	YES	YES

*Notes:* The t statistics in parentheses are based on robust standard errors clustered at the firm level. ^*^, ^**^, and ^***^ represent significance levels of 0.10, 0.05, and 0.01, respectively.

### Effects of financial constraint alleviation

Table 5 provides the results of the regression analysis testing the mediating role of SA in the relationship between PDP and TFP. In column (3), the results reveal an inverse relationship between PDP $\gamma_{1}$ =−0.024, p < 0.01). In column (4), TFP has a significantly negative coefficient for SA $\delta_{1}$ =−0.303, p < 0.01), and the magnitude of the direct effect of PDP $\delta_{1}$ =0.120, p < 0.01) decreases compared with the coefficient in the baseline regression. This suggests that SA partially mediates the relationship between PDP and TFP. We conduct a Sobel test to assess the magnitude of the mediating effect, resulting in a Z value of 2.702, which is significant at the 1% level and affirms the presence of the mediating effect of SA in the relationship between PDP and TFP. Therefore, H3 is supported.

### Effects of corporate disclosure enhancement

Table 5 provides the results of the regression analysis testing the mediating role of KV in the relationship between PDP and TFP. In column (5), the results reveal an inverse relationship between PDP and KV ($\gamma_{1}$ =0.073, p < 0.01). In column (6), TFP has a significantly negative coefficient for KV ($\delta_{1}$ =0.202, p < 0.01), and the magnitude of the direct effect of PDP on TFP ($\delta_{1}$ =0.112, p < 0.01) decreases compared with the coefficient in the baseline regression. These results indicate that KV partially mediates the relationship between PDP and TFP. We conduct a Sobel test to assess the magnitude of the mediating effect, resulting in a Z value of 5.122 that is significant at the 1% level and confirms the presence of the mediating effect of KV on the relationship between PDP and TFP. Therefore, H4 is supported.

### The reinforcing role of industry–university–research collaboration in increasing the impact of public digital procurement on corporate total factor productivity

Table 6 presents the regression results of IUR collaboration on corporate TFP. As evident from Table 6, the regression coefficients of IUR collaboration on corporate TFP remain consistently positive and significant. The results demonstrated in Model 4 reveal that after incorporating a series of control variables and controlling for both year and firm fixed effects, the coefficient of IUR is 0.048 and significant at the 5% level. This finding validates Hypothesis H5, suggesting that IUR collaboration significantly encourages enterprises to increase their TFP.

**Table 6:** The impact of IUR collaboration on corporate TFP.

Variables	Model 1	Model 2	Model 3	Model 4
**IUR**	0.321^***^ (0.050)	0.310^***^ (0.050)	0.093^***^ (0.025)	0.048^**^ (0.023)
**Cashflow**	2.556^***^ (0.060)	2.544^***^ (0.061)	1.048^***^ (0.058)	0.953^***^ (0.057)
**Lev**	1.491^***^ (0.021)	1.502^***^ (0.021)	0.470^***^ (0.054)	0.285^***^ (0.052)
**Growth**	0.277^***^ (0.010)	0.278^***^ (0.011)	0.246^***^ (0.010)	0.272^***^ (0.009)
**Board**	0.274^***^ (0.020)	0.295^***^ (0.021)	−0.001 (0.036)	0.071^**^ (0.034)
**TobinQ**	−0.080^***^ (0.003)	−0.076^***^ (0.003)	−0.043^***^ (0.005)	−0.020^***^ (0.005)
**Cap**	0.400^***^ (0.005)	0.396^***^ (0.005)	0.529^***^ (0.019)	0.377^***^ (0.021)
**_cons**	−0.306^***^ (0.081)	−0.298^***^ (0.082)	−1.175^***^ (0.292)	0.920^***^ (0.316)
**Obs**	26049	26049	25551	25551
**R** ^**2**^	0.428	0.431	0.899	0.910
**Firm effects**	NO	NO	YES	YES
**Year effects**	NO	YES	NO	YES

*Notes:* The t statistics in parentheses are based on robust standard errors clustered at the firm level. ^*^, ^**^, and ^***^ represent significance levels of 0.10, 0.05, and 0.01, respectively.

**Fig 3:**
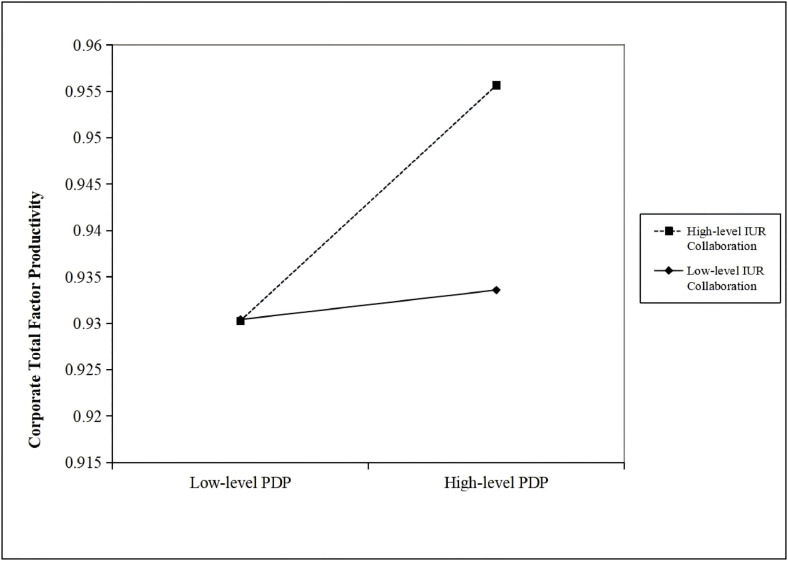
The enhancing effect of IUR collaboration.

Table 7 presents the enhancing effects of IUR collaboration on the relationship between PDP and TFP. As evident from Table 7, the regression coefficients of PDP, IUR, and their interaction term PDPIUR remain consistently positive and significant. The results demonstrated in Model 4 reveal that after incorporating a series of control variables and controlling for both year and firm fixed effects, the coefficient of the interaction term between PDPIUR is 1.385 and significant at the 1% level. To more clearly demonstrate the enhancing effect of IUR collaboration, this paper presents an enhancing effect diagram (see Fig 3). Fig 3 shows that for enterprises with existing IUR collaboration, the positive impact of PDP on corporate TFP is further strengthened. Moreover, this reinforcing effect is even more pronounced at higher levels of PDP. These findings validate Hypothesis H6.

**Table 7:** The enhancing effect of IUR collaboration.

Variables	Model 1	Model 2	Model 3	Model 4
**PDP**	0.459^***^ (0.075)	0.413^***^ (0.075)	0.213^***^ (0.038)	0.122^***^ (0.039)
**IUR**	0.327^***^ (0.050)	0.316^***^ (0.050)	0.095^***^ (0.024)	0.050^**^ (0.022)
**PDPIUR**	2.963^**^ (1.276)	2.910^**^ (1.273)	1.471^***^ (0.397)	1.385^***^ (0.471)
**Cashflow**	2.571^***^ (0.060)	2.559^***^ (0.061)	1.048^***^ (0.058)	0.953^***^ (0.057)
**Lev**	1.493^***^ (0.021)	1.504^***^ (0.021)	0.469^***^ (0.054)	0.285^***^ (0.052)
**Growth**	0.277^***^ (0.010)	0.278^***^ (0.011)	0.246^***^ (0.010)	0.272^***^ (0.009)
**Board**	0.276^***^ (0.020)	0.297^***^ (0.021)	0 (0.036)	0.072^**^ (0.034)
**TobinQ**	−0.080^***^ (0.003)	−0.076^***^ (0.003)	−0.042^***^ (0.005)	−0.020^***^ (0.005)
**Cap**	0.401^***^ (0.005)	0.397^***^ (0.005)	0.529^***^ (0.019)	0.377^***^ (0.021)
**_cons**	−0.329^***^ (0.081)	−0.319^***^ (0.082)	−1.182^***^ (0.292)	0.913^***^ (0.316)
**Obs**	26049	26049	25551	25551
**R** ^**2**^	0.429	0.432	0.899	0.910
**Firm effects**	NO	NO	YES	YES
**Year effects**	NO	YES	NO	YES

*Notes:* The t statistics in parentheses are based on robust standard errors clustered at the firm level. ^*^, ^**^, and ^***^ represent significance levels of 0.10, 0.05, and 0.01, respectively.

## 6. Further analysis

Digital transformation serves as a critical driver of corporate TFP growth. By enriching production factors, increasing division-of-labor efficiency, reducing labor costs, and optimizing human capital structures, it establishes a solid foundation for TFP improvement [17]. Examining the catalytic effect of PDP on corporate TFP, this study posits that digital transformation has a synergistic effect. The theoretical logic is that enterprises with advanced digital infrastructure can more effectively convert PDP resources into digital capability, thus fully unlocking policy dividends. Specifically, digital transformation facilitates TFP growth by internalizing technical specifications and data integration from procurement into production process optimizations while assimilating industry best practices during contract fulfillment [42]. If this hypothesis holds, then the primary effect of PDP on TFP should increase with increasing corporate digital capabilities.

To empirically test this synergy between PDP and digital transformation, we adopted the methodology of [43] to construct a corporate digital transformation intensity index (DIG) based on textual analysis of annual reports. An interaction term (PDPDIG) was incorporated into the econometric model. The results in Table 8 Model 4 show that, after controlling for relevant variables and absorbing firm and year fixed effects, the interaction term coefficient is 0.055, which is statistically significant at the 5% level. This finding indicates complementary effects between PDP and corporate digital transformation. Enterprises with higher DIG levels leverage digital contract resources more efficiently to enhance digital capabilities, achieving a multiplying effect on TFP growth.

**Table 8:** Further analysis.

Variables	Model 1	Model 2	Model 3	Model 4
**PDP**	−0.227 (0.207)	−0.275 (0.206)	−0.001 (0.090)	−0.057 (0.090)
**DIG**	0.063^***^ (0.003)	0.060^***^ (0.003)	0.059^***^ (0.005)	0.028^***^ (0.005)
**PDPDIG**	0.113^**^ (0.057)	0.119^**^ (0.057)	0.063^**^ (0.027)	0.055^**^ (0.027)
**Cashflow**	2.674^***^ (0.060)	2.660^***^ (0.060)	1.054^***^ (0.059)	0.958^***^ (0.058)
**Lev**	1.511^***^ (0.021)	1.516^***^ (0.021)	0.445^***^ (0.054)	0.283^***^ (0.052)
**Growth**	0.275^***^ (0.010)	0.273^***^ (0.011)	0.247^***^ (0.010)	0.272^***^ (0.009)
**Board**	0.305^***^ (0.020)	0.317^***^ (0.020)	−0.003 (0.035)	0.066^*^ (0.034)
**TobinQ**	−0.082^***^ (0.003)	−0.079^***^ (0.003)	−0.041^***^ (0.005)	−0.020^***^ (0.005)
**Cap**	0.407^***^ (0.005)	0.404^***^ (0.005)	0.513^***^ (0.019)	0.377^***^ (0.021)
**_cons**	−0.565^***^ (0.082)	−0.547^***^ (0.082)	−1.009^***^ (0.290)	0.889^***^ (0.315)
**Obs**	26049	26049	25551	25551
**R** ^**2**^	0.437	0.439	0.901	0.911
**Firm effects**	NO	NO	YES	YES
**Year effects**	NO	YES	NO	YES

*Notes:* The t statistics in parentheses are based on robust standard errors clustered at the firm level. ^*^, ^**^, and ^***^ represent significance levels of 0.10, 0.05, and 0.01, respectively.

## 7. Conclusions

### Theoretical contribution

From the perspectives of demand-induced innovation theory and asymmetric information theory, this study employs a comprehensive panel dataset of Chinese listed firms (2015–2023) to investigate the relationship between PDP and corporate TFP. Our findings provide robust evidence that PDP implementation significantly increases corporate TFP, a conclusion that is validated through rigorous endogeneity and robustness tests. This study delves into the underlying mechanisms, pinpointing three pivotal pathways: DT innovation, financing constraint alleviation, and information disclosure. Furthermore, we reveal the positive moderating effect of IUR collaboration on this relationship. These findings enrich existing theoretical frameworks by offering nuanced insights from the perspectives of technological upgrading and resource allocation. Specifically:

(i) While the literature has comprehensively examined the impact of digital government policies on corporate development [13], we adopt a public procurement lens to reveal how PDP functions as a policy instrument under such policies, providing a complementary perspective. We innovatively construct a PDP index by integrating LLM techniques with data from Chinese listed firms (2015–2023) and textual information from the China Government Procurement Network, forming panel data for empirical analysis. This approach effectively mitigates the measurement errors inherent in traditional policy metrics [44], offering microlevel evidence for evaluating the economic effects of PDP.
(ii) We advance the theoretical frontier of TFP determinants. Prior studies predominantly emphasized supply-side drivers of TFP [10,14], with limited attention paid to demand-side factors. We shift the focus to public procurement—a classical demand‒pull instrument—and rigorously investigate its relationship with TFP. Our findings demonstrate that PDP significantly increases TFP, providing new perspectives and complementary insights into the factors influencing TFP. Moreover, this finding lends support to the theoretical discovery of [45] that, from a supplier sustainability perspective, public procurement can significantly enhance suppliers’ sustainable development capacity and increase corporate TFP.
(iii) We advance the microeconomic analysis of PDP. Although prior research has demonstrated positive effects of overall public procurement on corporate productivity [12], it has failed to capture the digital attributes of procurement contracts. In contrast, we innovatively isolate and focus on digital procurement orders, constructing a dedicated PDP index that overcomes the neglect of digital characteristics in traditional studies. This perspective significantly extends the research scope of [46,47] with regard to innovation-oriented and green procurement. Our empirical results indicate that PDP exerts consistently significant positive effects on corporate TFP, in contrast with the U-shaped relationship reported by [12]. We argue that by specifically focusing on high-tech digital procurement (rather than overall government procurement), PDP continuously improves firm efficiency without the initial negative effects potentially present in traditional procurement policies. These findings contribute to a more comprehensive and balanced understanding of the heterogeneous impacts of different types of government procurement.
(iv) Furthermore, this study uncovers the intrinsic mechanisms (“black box”) through which PDP enhances corporate TFP. While existing research widely recognizes the significant potential of PDP as an emerging policy tool, its internal mechanisms remain insufficiently explained [4]. We identify and empirically test three critical mediating mechanisms: DT innovation, financing constraint alleviation, and information disclosure. Among these, DT innovation and financing constraint alleviation align with the findings of [2], further validating the robustness and universality of these mechanisms in the PDP context. Moreover, this study adds a novel mechanism, information disclosure, systematically clarifying the joint operation of multiple mechanisms. In doing so, this study effectively extends the understanding of PDP mechanisms in the literature.

### Management implications

From a practical perspective, our findings offer significant insights for government digital procurement decisions and corporate development planning.

First, considering that the scale of government digital procurement in China still has substantial room for growth and must address the practical needs of enterprise development, the government should focus on expanding procurement in the digital domain. During implementation, the accurate identification of procurement attributes and enterprise characteristics is essential for enhancing policy effectiveness through differentiated strategies. Simultaneously, accelerating the development of digital government and the deployment of computing infrastructure will promote the efficient flow of key data elements. This will not only significantly improve the TFP of enterprises but also contribute to the inclusive distribution of digital dividends.

Second, the government should construct a policy portfolio toolkit to drive collaborative innovation between digital procurement and enterprise transformation. By designing differentiated policies to support enterprises’ digital transformation—combining direct incentives such as tax reductions and R&D subsidies with indirect support measures such as financing assistance—a synergistic policy force can be formed. This leverages procurement demand to steer enterprises, thus enhancing policy efficacy and fostering corporate growth.

Third, IUR collaboration plays a vital role in the R&D and application processes of government digital procurement. The government must lead in establishing collaborative platforms that integrate research resources, enterprise scenarios, and government procurement needs. These platforms should actively advance PDP projects to break through technological bottlenecks, accelerate the achievement transformation process, and concurrently focus on innovating cooperation mechanisms while optimizing the collaborative environment. This will provide more efficient and convenient resource matching and achievement transformation services for participating enterprises and research institutions.

### Limitations and future studies

Notably, our study has several limitations. First, the development of the digital economy has a pronounced long-term and dynamic nature [48]. The data period used in this study (2015–2023) may not fully capture the long-term impact of PDP on TFP. Therefore, future research could further extend the time span, for example, by tracking firm-level microdata (including numerous SMEs) over several decades, to comprehensively capture the long-term dynamic impact of PDP on corporate productivity. On the other hand, although this study innovatively constructs a PDP index via LLM technology, this approach may still suffer from an insufficient capture of data dimensions and policy implementation details. Key factors that influence policy effectiveness—such as the actual implementation efficiency of government procurement contracts and the frequency of firm‒government interactions—remain inadequately examined [49,50]. We recommend that subsequent research collect further interview and survey data from local governments or enterprises, gather and analyze government procurement contract audit documents, and integrate real-time transaction data from government e-procurement platforms. These approaches more comprehensively capture the implementation heterogeneity and granular details of PDP policies, thus increasing the precision and completeness of policy effect evaluations.

## Supporting information

S1 File Code. (DTA)

S2 File Data. (TXT)

S3 File Python sript. (PY)

S4 File Keyword repository for PDP. (TXT)
